# Salmon Aquaculture and Antimicrobial Resistance in the Marine Environment

**DOI:** 10.1371/journal.pone.0042724

**Published:** 2012-08-08

**Authors:** Alejandro H. Buschmann, Alexandra Tomova, Alejandra López, Miguel A. Maldonado, Luis A. Henríquez, Larisa Ivanova, Fred Moy, Henry P. Godfrey, Felipe C. Cabello

**Affiliations:** 1 Centro i∼mar, Universidad de Los Lagos, Puerto Montt, Chile; 2 Department of Microbiology and Immunology, New York Medical College, Valhalla, New York, United States of America; 3 Department of Pathology, New York Medical College, Valhalla, New York, United States of America; University of Otago, New Zealand

## Abstract

Antimicrobials used in salmon aquaculture pass into the marine environment. This could have negative impacts on marine environmental biodiversity, and on terrestrial animal and human health as a result of selection for bacteria containing antimicrobial resistance genes. We therefore measured the numbers of culturable bacteria and antimicrobial-resistant bacteria in marine sediments in the Calbuco Archipelago, Chile, over 12-month period at a salmon aquaculture site approximately 20 m from a salmon farm and at a control site 8 km distant without observable aquaculture activities. Three antimicrobials extensively used in Chilean salmon aquaculture (oxytetracycline, oxolinic acid, and florfenicol) were studied. Although none of these antimicrobials was detected in sediments from either site, traces of flumequine, a fluoroquinolone antimicrobial also widely used in Chile, were present in sediments from both sites during this period. There were significant increases in bacterial numbers and antimicrobial-resistant fractions to oxytetracycline, oxolinic acid, and florfenicol in sediments from the aquaculture site compared to those from the control site. Interestingly, there were similar numbers of presumably plasmid-mediated resistance genes for oxytetracycline, oxolinic acid and florfenicol in unselected marine bacteria isolated from both aquaculture and control sites. These preliminary findings in one location may suggest that the current use of large amounts of antimicrobials in Chilean aquaculture has the potential to select for antimicrobial-resistant bacteria in marine sediments.

## Introduction

It is believed that aquaculture will constitute the source of over more than half of the seafood consumed in the world in coming years because of the collapse of natural fisheries [1]. However, this optimistic view needs to be tempered by increasing information suggesting such expansion may be unsustainable as aquaculture generates untoward effects such as habitat destruction, eutrophication and environmental contamination with chemicals and antimicrobials [2]. The therapeutic, growth-promoting and prophylactic use of antimicrobials was introduced into agricultural practice in the 1940s and became widespread in Europe and the United States [3]–[6]. Antimicrobial resistance in antimicrobial-fed animals was soon noted [7], concerns about the possibilities of transmission of this resistance to human pathogens followed shortly thereafter [6], [8], [9], and indeed, has been demonstrated to occur [10], [11]. Voluntary and legislated bans on the use of antimicrobials as growth promoters in the member states of the European Union since the 1990s have been associated with a marked decrease in antimicrobial usage without negative impacts on productivity in fowl and swine [8], [12], [13].

Salmon aquaculture is an exponentially growing industry worldwide, particularly in two countries – Norway and Chile [14], [15]. In Chile, this growth has been accompanied by major mortalities of salmon reared in net pens. These can reach 50% of production under some conditions with ensuing large economic losses [16], [17]. This growth has triggered concerns regarding many environmental issues, particularly because large amounts of chemotherapeutics and antimicrobials in the feed readily pass into the marine environment and potentially alter bacterial biodiversity [2], [18]–[22]. Because the use of vaccines to prevent bacterial diseases in fish is limited [16], this in turn has led to increased use of therapeutic and prophylactic antimicrobials [23]–[25]. Conservative estimates suggest that approximately 950 metric tons of quinolones were used in salmon aquaculture in Chile between 2000 and 2008, and approximately 1500 metric tons of tetracycline and 478 metric tons of florfenicol were used for this purpose between 2000 and 2007 [23]–[25].

Antimicrobial agents are usually administered to salmon mixed with food [19], [22]. Uningested food and fish feces containing unabsorbed antimicrobials and secreted antimicrobial metabolites in the water and sediment in the environment of salmon farming sites often retain their antimicrobial activity and can remain in the aquatic environment for variable periods of time depending on their initial concentrations, biodegradability, and physical and chemical characteristics [19], [26]–[28]. Such materials can select for antimicrobial resistant bacteria in the sediment and water column and can often influence microbial diversity not only by eliminating susceptible bacteria but also by acting on other susceptible microorganisms such as microalgae [22], [26], [29], [30].

Selection of antimicrobial-resistant bacteria in the marine environment could have detrimental impacts on piscine and human health by facilitating transfer of antimicrobial resistance genetic determinants from environmental marine microbes to fish pathogens and terrestrial bacteria including human and animal pathogens [19], [22], [23], [31]. It is clearly evident that bacteria from marine and terrestrial ecosystems can share antimicrobial resistance genes and that some emerging antimicrobial resistance genes in human pathogens may have an aquatic bacterial origin [32]–[35]. For example, the fish pathogen *Yersinia ruckerii*, the cause of enteric redmouth disease, shares an antimicrobial resistance plasmid and antimicrobial resistance genes with the plague bacillus, *Yersinia pestis* [36]. This sharing of movable genetic elements and antimicrobial resistance genes between bacteria of different ecological niches potentially endangers treatment of human patients [22], [32]–[34], [36]. Such genetic and epidemiological findings strongly suggest that the aquatic and terrestrial ecosystems are not isolated regarding the dissemination of antimicrobial resistance genes among their bacterial populations, probably as the result of horizontal gene transfer [22], [37].

The high level of antimicrobial use in salmon aquaculture in Chile could have negative impacts on environmental biodiversity and terrestrial animal and human health by selecting for bacteria in the marine environment containing antimicrobial resistance genes. We therefore compared numbers of culturable bacteria and antimicrobial resistant bacteria for three antimicrobials used extensively in Chilean salmon aquaculture (oxytetracycline, oxolinic acid, and florfenicol) in the marine sediment adjacent to salmon aquaculture pens and at a control site 8 km distant with no observed aquaculture or other human activities.

## Results

### Antimicrobials in sediment samples from aquaculture and control sites

**Figure 1:**
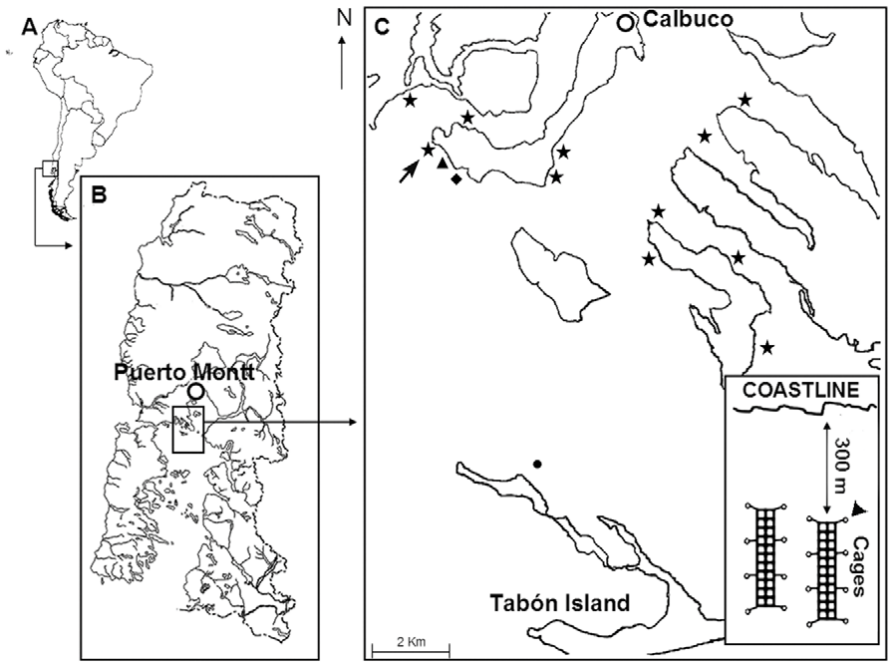
Locations of aquaculture and sites of sampling in the Calbuco archipelago, Chile. Salmon farming sites are indicated by stars. The “aquaculture site” sampled in the present study (arrowhead, inset) was 20 m from the salmon farm indicated by arrow. Other sites sampled in the present study were located 0.5 km (solid triangle), 1 km (solid diamond) and 8 km (solid circle) from the aquaculture site. The latter site was off the coast of Tabón Island, an island with no aquacultural activities or other human activity, and is referred to as the “control site” in the text.

Traces of flumequine, a fluoroquinolone antimicrobial, were present in four sediment samples at the aquaculture site (Fig. 1): two in December, 2008, and two in January, 2009. Flumequine was also present in four sediment samples from the control site, 8 km from the aquaculture site (Fig. 1): one in December, 2008, two in January, 2009 and one in April, 2009. Oxytetracycline, oxolinic acid, and florfenicol were not detected in any of the 36 total samples examined (data not shown).

### Culturable bacteria in sediment samples from aquaculture and control sites

**Figure 2:**
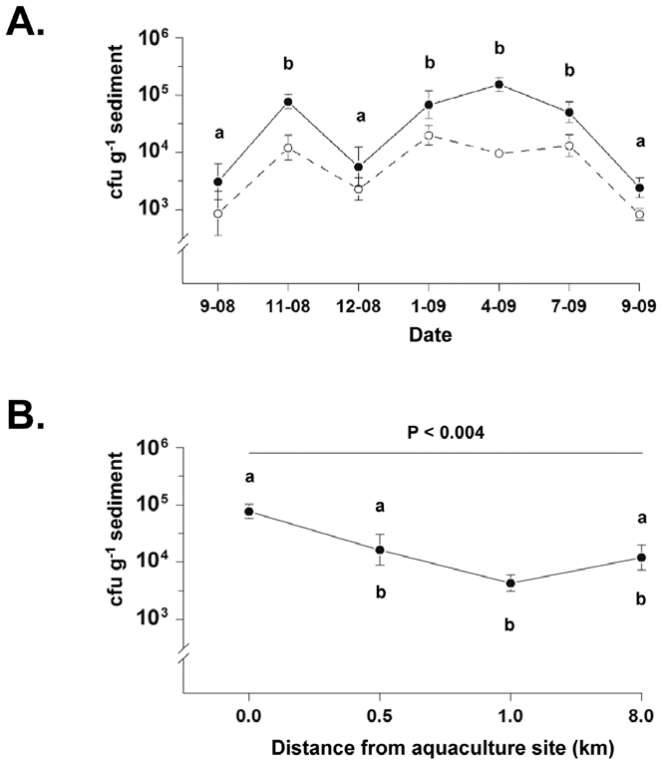
Culturable bacteria in sediment samples at aquaculture and control sites taken at various time points. A. Colony forming units (cfu) g^−1^ sediment (mean ± SE) in samples taken from September, 2008, to September, 2009, were significantly higher at the aquaculture site (closed circles) than at the control site (open circles) at all time points (P<0.001); different lower case letters indicate significant differences (P<0.05). A total of 66 samples were taken, 33 from the aquaculture site and 33 from the control site. B. Cfu g^−1^ sediment (mean ± SE) taken in November, 2008, at the aquaculture site (0.0 km) and at sites 0.5, 1.0 and 8.0 km (control site) distant from it. Aquaculture and control sites correspond to sites shown in Fig. 2A for this date; five samples were taken from each additional site studied. Different lower case letters indicate significant differences (P<0.05, see text for details of statistical analysis).

The total numbers of culturable bacteria in sediments from aquaculture and control sites varied significantly over the course of a year (P<0.001, two-way ANOVA, rank transformed data), fluctuating between approximately 1×10^3^ and 1×10^5^ colony forming units (cfu) g^−1^ (Fig. 2A). There were highly significant differences in culturable bacterial numbers between aquaculture and control sites over the entire study period (P<0.001, rank transformation test), with bacterial numbers significantly higher in late spring (November, 2008) and high summer through winter (January through July, 2009) than at other times (P<0.05, Student-Newman-Keuls post-test). Sensitivity analyses using only data consistent with a dilution series or using all 258 data points produced similar results. Results obtained using a standard two-way ANOVA of log-transformed data were also consistent with this non-parametric analysis.

Sampling of sediments at intermediate distances between aquaculture (0.0 km) and control (8.0 km) sites in November, 2008 (Fig. 2B), revealed significant differences in numbers of culturable bacteria (P<0.001, two-way ANOVA, rank transformed data) which did not decrease until 1 km from the aquaculture site (P<0.05, Student-Newman-Keuls post-test). Sensitivity analyses using only data consistent with a dilution series or using all 258 data points produced similar results. Results obtained using ANOVA of log-transformed data were also consistent with non-parametric analysis.

### Antimicrobial-resistant bacteria in sediment samples from aquaculture and control sites

**Figure 3:**
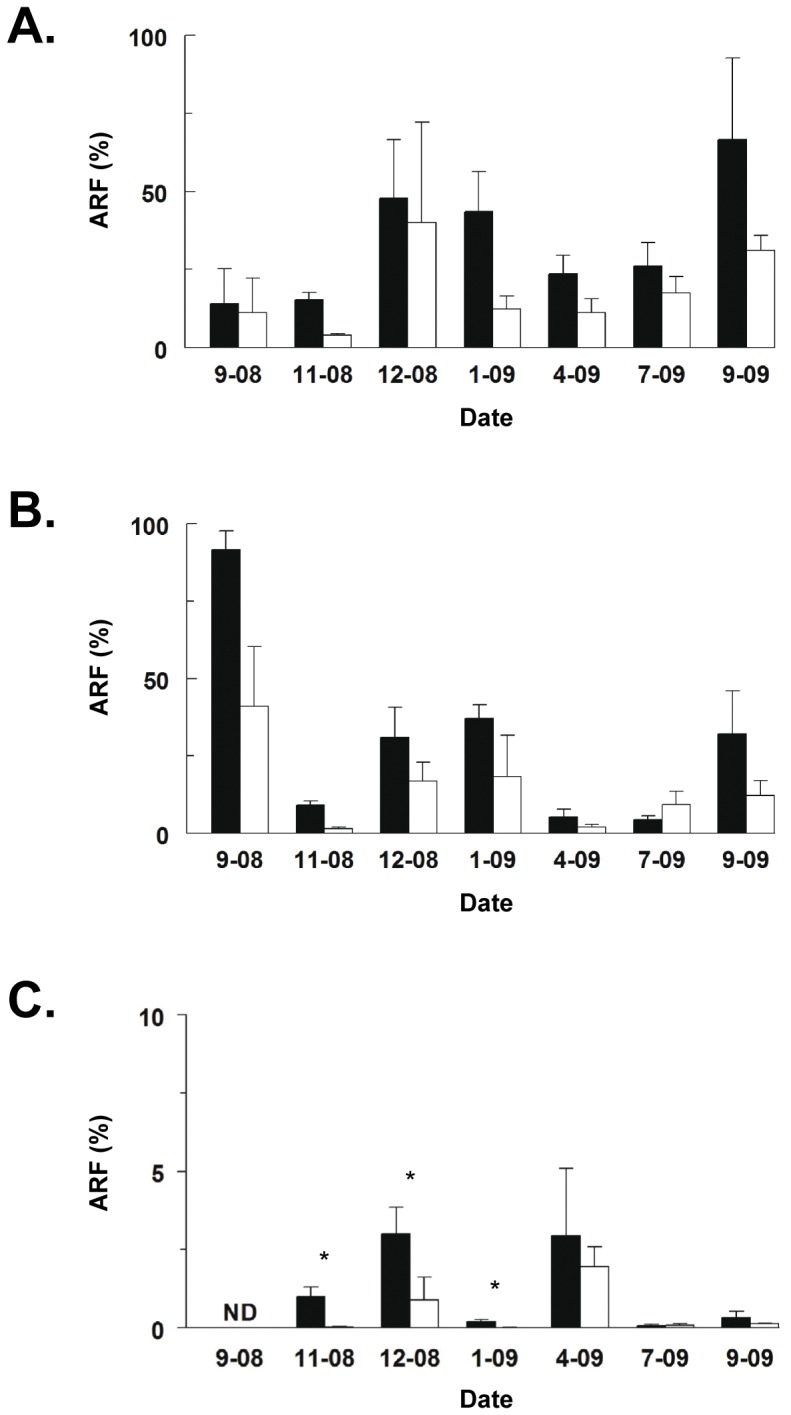
Antimicrobial resistant bacteria in sediment samples from aquaculture and control sites. Antimicrobial resistance fraction (ARF) (mean ± SE) of culturable bacteria to (A) oxytetracycline and (B) oxolinic acid in sediments from aquaculture (solid bars) and control (open bars) sites from September, 2008, to September, 2009, were significantly different between aquaculture and control sites over the entire study period (P<0.001). ARF to (C) florfenicol in sediments from aquaculture and control sites were significantly different only in November, 2008, December, 2008, and January, 2009. *, P<0.05, see text for details of statistical analysis. A total of 66 samples were taken, 33 from the aquaculture site and 33 from the control site.

Measurements of the antimicrobial resistant fraction (ARF) of bacteria cultured from aquatic sediments are useful for comparing changes in antimicrobial resistance in this environment [38]. ARF to oxytetracycline (Fig. 3A) and oxolinic acid (Fig. 3B) were significantly different between aquaculture and control sites over the entire period of study (P<0.001, two-way ANOVA, rank transformed data). For both these antimicrobials, there was no significant interaction between time of year and nature of the study site (aquaculture or control). ARF to oxytetracycline was significantly lower during spring, 2008 (September-November, 2008) than during the following summer (December, 2008–January, 2009), before rising significantly the following spring (September, 2009) (P<0.05, Student-Newman-Keuls post-test). ARF to oxolinic acid (Fig. 3B) also was significantly higher in early spring both years (September, 2008; September, 2009) than in late spring, fall and winter (November, 2008; April, 2009: July, 2009), before rising to intermediate levels in high summer (December, 2008; January, 2009) (P<0.05, Student-Newman-Keuls post-test). Although ARF to florfenicol also varied significantly throughout the year (Fig. 3C), the interaction of time and study site was significant (P<0.002, two-way ANOVA, rank transformed data), and significant differences between aquaculture and control sites were only seen in late spring and high summer (November, 2008, through January, 2009) (P<0.05, Student-Newman-Keuls post-test). Sensitivity analyses using only data consistent with a dilution series or using all 258 data points produced similar results. Results obtained with parametric analysis of log-transformed data were again consistent with this non-parametric analysis.

**Figure 4:**
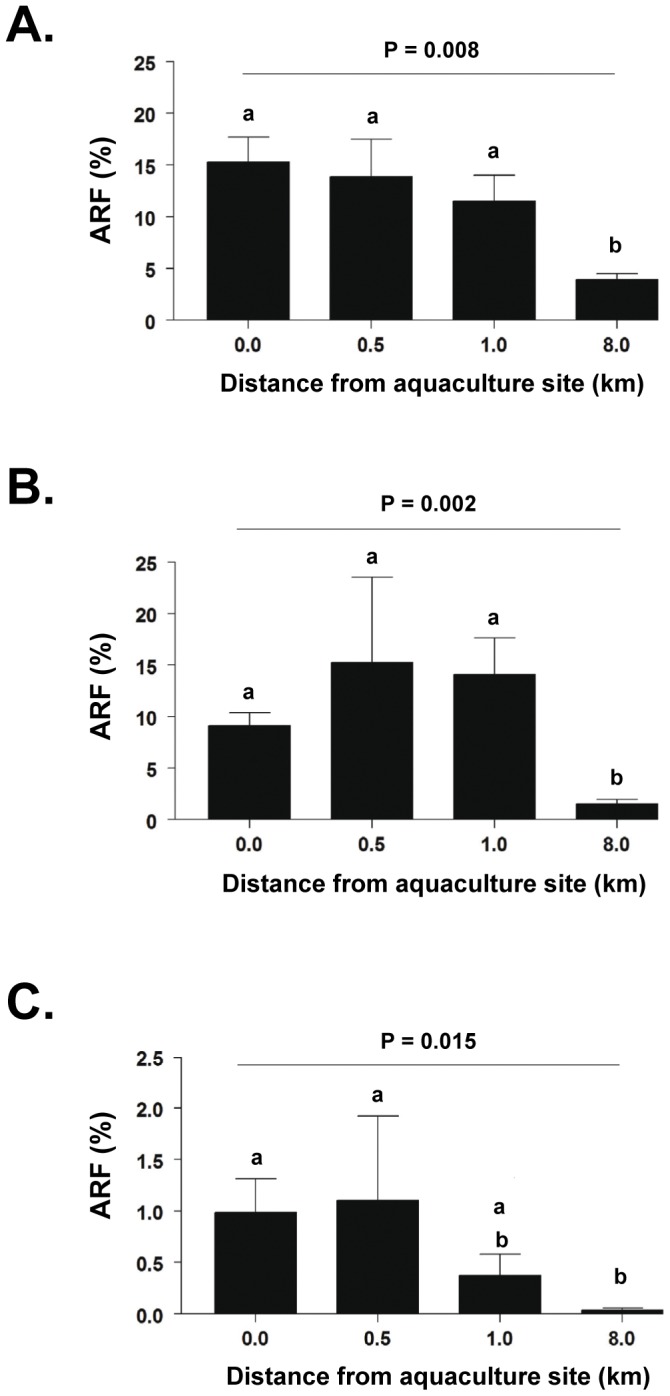
Variation in ARF to selected antimicrobials with distance from aquaculture site. ARF (mean ± SE) in November, 2008, to (A) oxytetracycline, (B) oxolinic acid, and (C) florfenicol in sediments at aquaculture site (0.0 km) and at sites 0.5, 1.0 and 8.0 km (control site) distant from it. ARF for aquaculture and control sites correspond to ARF shown in Fig. 3 for this date. Five samples were taken from each additional site studied. ARF to each antimicrobial were significantly greater at the aquaculture than at the control site (probabilities indicated for each antimicrobial). Different lower case letters within each panel indicate significant differences between ARF (P<0.05, see text for details of statistical analysis).

Sampling in November, 2008, at intermediate distances between aquaculture and control sites revealed that ARF to oxytetracycline (Fig. 4A), oxolinic acid (Fig. 4B), and florfenicol (Fig. 4C) showed significant decreases from aquaculture to control sites for all three antimicrobials (oxytetracycline, P = 0.008; oxolinic acid, P = 0.002; florfenicol, P = 0.015, two-way ANOVA, rank transformed data). In the case of oxytetracycline and oxolinic acid (Figs. 4A, 4B), ARF was only significantly lower 8 km from the aquaculture site (P<0.05, Student-Newman-Keuls post-test). Significant elevations of ARF to florfenicol were maintained 0.5 km from the aquaculture site, but were significantly lower by 1 km (Fig. 4C). Sensitivity analyses using only data consistent with a dilution series or using all 258 data points yielded similar results. Results from parametric analysis of log-transformed data were again consistent with this non-parametric analysis.

### Detection of antimicrobial resistance genes in bacteria from aquaculture and control sites

**Figure 5:**
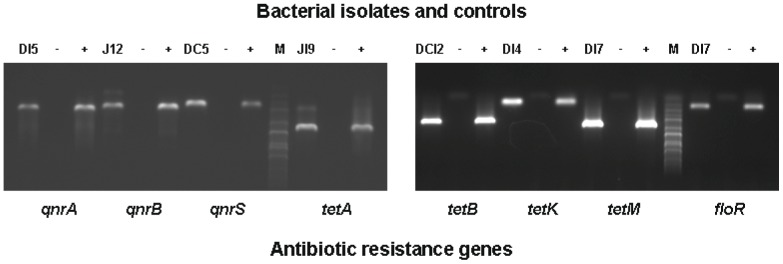
Antimicrobial resistance genes in unselected marine bacterial isolates and controls. Detection of antimicrobial resistance genes in bacteria cultured from marine sediments obtained from December, 2008, to January, 2009. *qnr*, *tet* and *floR* genes were detected by PCR as described in Material and Methods with primers in Table 3. D15, J12, DC5, J19, DC12, D14 and D17 are bacterial isolates from sediment. −, negative control (*E. coli* DH5α). +, positive controls (Table 3). M, molecular weight markers.

The presence of culturable antimicrobial-resistant bacteria in sediments from aquaculture and control sites suggested the presence of antimicrobial resistance determinants in these bacteria. PCR confirmed the presence of genes mediating resistance to oxytetracycline, oxolinic acid, and florfenicol in bacteria from sediment samples that had not been selected for antimicrobials. Plasmid-mediated quinolone resistance (PMQR) genes were studied because these plasmid-mediated resistances are potentially transmissible and some of them appear to originate from aquatic bacteria [32], [33], [39], [40]. Moreover, they have recently begun to disseminate among terrestrial animal and human pathogens and are readily detected by PCR [40]. Unselected isolates of bacteria from aquaculture and control sites (24 from each site) contained plasmid-encoded genes for resistance to quinolones, including *qnrA*, *qnrB*, *qnrS*, *oqxA* and *aac(6′)-Ib-cr* (Fig. 5, Table 1). Several of these bacteria also harbored *tetA*, *tetB*, *tetK*, *tetM*, and *floR* genes (Fig. 5, Table 1). Some bacteria harbored multiple antimicrobial resistant determinants (Table 2). The combination of PMQR and tetracycline resistance genes was the most frequent (8), followed by PMQR and florfenicol resistance genes (3) and finally PMQR, tetracycline and florfenicol resistance genes (3). Several isolates also generated a positive signal (confirmed by DNA sequencing) for the *int1* gene encoding integrase 1 (Table 1), suggesting the presence of type 1 integrons in these bacteria [41]–[45].

**Table 1:** Antimicrobial resistance genes present in marine sediment bacteria from aquaculture and control sites in Chile.

		Antimicrobial resistance genes to													
		Tetracycline				Quinolones								Florfenicol	
Site	No. of Strains	*tetA*	*tetB*	*tetK*	*tetM*	*qnrA*	*qnrB*	*qnrC*	*qnrD*	*qnrS*	*qepA*	*oqxA*	*aac(6′)-Ib-cr*	*floR*	*int1*
Aquaculture	24	4	5	5	2	2	1	0	0	3	0	4	4	4	3
Control	24	3	7	4	0	2	1	0	0	5	0	3	1	2	1

**Table 2:** Bacterial isolates identified by 16S rRNA gene sequence analysis.

Species	Genes	Compared to GenBank No.	% of identity	Site of isolation
*Sporosarcina* sp.	*tetK, floR, qnrA, qnrS*	FJ425906.1	>99	Aquaculture
*Arthrobacter* sp.	*qnrA, tetB*	EF550164.1	>99	Aquaculture
*Sporosarcina* sp.	*oqxA, aac(6′)-1b-cr*	EU204977.1	>99	Aquaculture
*Arthrobacter* sp.	*qnrS, tetA*	JF799958.1	>99	Aquaculture
*Vibrio* sp.	*qnrB, tetK*	DQ146994.1	>99	Aquaculture
*Pseudoalteromonas* sp.	*qnrA, tetB*	FJ497709.1	>99	Control
*Vibrio* sp.	*qnrS*	EU195936.1	>99	Control
*Vibrio* sp.	-	JN128258.1	>99	Control

### Species identification of bacteria harboring antimicrobial resistance genes from aquaculture and control sites

PCR amplification of 16S rRNA genes in eight bacterial isolates from the aquaculture site identified two isolates of *Sporosarcina* sp., two isolates of *Arthrobacter* sp. and one isolate *Vibrio* sp. Bacterial isolates from the control site included one *Pseudoalteromonas* sp. isolate and two isolates of *Vibrio* sp. (Table 2). The 16S rDNA sequences of these amplicons were >99% identical to those in GenBank (E value of 0.0) (Table 2). These observations are not consistent with the possibility that the bacteria in which antimicrobial resistance genes were present were human and terrestrial animal pathogens contaminating Chilean coastal waters [46], [47].

## Discussion

We studied marine sediments from two sites in the Calbuco Archipelago in southern Chile to determine numbers of culturable and antimicrobial-resistant bacteria they contained. One was a site 20 m from a salmon aquaculture facility (Fig. 1), the other, off the coast of an island 8 km distant from the aquaculture site, the only local island with no aquaculture activities, few human dwellings, and no water sources or discharges of solids into the sea (Fig. 1). This latter site was thus expected to be pristine and a suitable control site. Surprisingly, residues of flumequine, a quinolone with potential cross resistance with oxolinic acid, were present in sediments at both sites, most likely carried there by marine currents from the many other aquaculture sites in the area that use this antimicrobial and that have been in operation over the past 10 years (Fig. 1) [23]–[25]. Flumequine has been used as widely as oxolinic acid in aquaculture in Chile; approximately 548 metric tons were used between 2000 and 2007 [23]–[25]. Although the control site was not as pristine as it was originally thought to be, sediments from the aquaculture site still contained significantly larger numbers of culturable bacteria than sediments from the control site (Fig. 2A), with increased bacterial numbers being present up to 1 km from the aquaculture site (Fig. 2B). These findings essentially confirm previous reports on the ability of aquaculture activities to increase culturable bacterial numbers [48], [49]. Sediments from the aquaculture site also contained increased numbers of culturable bacteria resistant to tetracycline, oxolinic acid and florfenicol; this effect persisted for distances up to 1 km from the aquaculture site (Fig. 4). The presence of flumequine residues in the sediment from the apparently pristine control site (Fig. 1) and significant antimicrobial resistance at distances up to 1 km from the aquaculture site (Fig. 4) suggest that excessive use of antimicrobials in salmon aquaculture sites may not only have an effect on marine sediments directly under and close to aquaculture pens but also at some distance from where these activities take place as a result of transport by water currents of both unchanged antimicrobials and their antimicrobially active metabolites. Previous work has not detected antimicrobials beyond 30 to 50 m from the aquaculture site but the amounts of antimicrobials used in those situations were a fraction of those used in Chile [26], [29], [50]–[52]. This suggests that the size of the area impacted by aquacultural activities with regard to antimicrobial resistance is related to the amounts of antimicrobials used in these activities.

Obtaining accurate counts of culturable bacteria in marine sediments is complicated by incomplete dispersal of particulates and their attached bacteria. This incomplete dispersal leads to erratic values in dilution series. Although clinical studies frequently employ various criteria to ensure the quality of data to be analyzed [53], [54], our study is one of the first if not the first in this area to use explicit criteria to assure the quality of the data to be analyzed. The fact that similar conclusions were obtained in multiple sensitivity analyses confirms the validity of this approach.

It has been suggested that the significantly larger numbers of culturable antimicrobial-resistant bacteria demonstrable in sediment of aquaculture sites relative to control sites may be the result of changes produced by excess organic matter passing into the environment from uningested fish food and feces rather than from antimicrobial use per se [55]–[57]. Unfortunately, there is no experimental evidence to support this hypothesis. It is difficult to develop a scenario based on current concepts of microbial genetics and physiology that could explain preferential stimulation of growth of antimicrobial-resistant bacteria by organic matter alone unless this matter also contained other chemical entities such as metal ions, disinfectants or metabolites that could co-select for metabolite utilization and ubiquitous antimicrobial resistance genes linearly integrated in mobile genetic units such as plasmids, transposons and integrons throughout Bacteria and Archea [58], [59].

An important limitation of this research is the lack of information concerning the use and frequency of application of antimicrobials during aquaculture activities before and during this study. This stems from the proprietary nature of this information and from the general lack of publicly available and well-organized data on antimicrobial use in the Chilean aquaculture industry. This dual lack critically limits our ability to relate antimicrobial use to the observed relative increase in antimicrobial-resistant bacteria and might also be responsible for our failure to detect antimicrobials other than flumequine in the sediments. The marked increase in antimicrobial-resistant bacteria in the spring and summer when activity at aquaculture sites customarily increases, the presence of flumequine residues and the increased ARF to antimicrobials known to be heavily used in this industry [23] does however suggest a possible relationship between them. It has been postulated that antimicrobials administered to fish by food do not remain in the sediment, thus decreasing their ability to exert selective pressure upon antimicrobial-resistant bacteria [51], However, the present and previous work indicate that antimicrobials remain in the sediment at concentrations able to exert selective pressure there [52], [60], [61].

Approximately half of unselected culturable marine bacterial species from both aquaculture and control sites harbored antimicrobial resistance genes (Table 1); the antimicrobial resistances detected in these bacteria are probably mediated by these genes. Because *tetM* tetracycline resistance gene and other antimicrobial resistance genes have been demonstrated in ancient (30,000 years before the present) bacterial DNA extracted from terrestrial permafrost in Alaska [62], the effect of antimicrobial use in salmon aquaculture on marine sediments is most likely restricted to selecting those bacteria able to survive in their presence. However, the numerically similar frequencies of antimicrobial resistance genes at both sites is certainly consistent with the presence of antimicrobial residues at both sites, and again suggests that the control site was not as pristine as it was originally thought to be.

There are several caveats regarding the observed bacterial resistance phenotypes and genotypes. Because we only sequenced three amplicons for the *aac(6′)-Ib-cr* gene, we cannot be certain that the five amplicons detected have the mutation which mediates quinolone acetylation [63]. Furthermore, oxytetracycline, oxolinic acid, and florfenicol resistance phenotypes can be encoded by a multiplicity of chromosomal and plasmid genes and not only by the ones studied in the present work [40], [64]–[66]. Because we did not perform an exhaustive investigation of alternative antimicrobial resistance genes for quinolone, tetracyclines and chloramphenicol, did not search for the presence of genes mediating resistance to other antimicrobials, and studied only culturable bacteria, we are probably underestimating the resistome present in marine bacteria at the aquaculture and control sites. This underestimation could lower the chance of detecting any differences regarding these genes between these sites. Interestingly, a few of the strains studied also harbored an integron type 1, a genetic element usually associated with multiple antimicrobial resistance cassettes and known to be present in bacteria from aquatic sediments impacted by human activity [41]–[44].

Bacteria from the marine environment where salmon aquaculture takes place contain antimicrobial resistance genes towards antimicrobials used extensively in this activity. This confirms previous work indicating that plasmid-mediated quinolone resistance genes are present in aquatic bacteria [32], [33], [39] and that these aquatic bacteria could well be the original source for dissemination of these determinants in human pathogens [22], [32], [33], [39]. Such bacteria will have a selective advantage after introduction of antimicrobials into their environment [22], [31], [37], [41]. The relevance of increased antimicrobial-resistant bacteria in sediments of salmon aquaculture sites for the emergence of antimicrobial resistance in fish and human pathogens is unknown [22], [23], [56], [57]. The significant increase in antimicrobial-resistant bacterial populations to oxytetracycline, oxolinic acid, and florfenicol in aquaculture sediments suggests they could be a potential source for antimicrobial resistance genes in fish and human pathogens as a result of horizontal gene transfer [18], [22], [23], [36], [67], [68]. This problem could be exacerbated in Chile because of major contamination of seawater with antimicrobial-resistant animal and human pathogens [46], [47]. Horizontal gene transfer of antimicrobial resistance genetic elements and mutagenesis may also be stimulated by microbial stress triggered by the presence of sub-inhibitory concentrations of antimicrobials such as flumequine in the sediment [68]–[70].

The present preliminary study in a single salmon aquaculture and a single control site suggests, as has been previously demonstrated, that salmon aquaculture activities in Chile have the potential to alter concentrations of culturable bacteria in marine sediments and increase the proportion of antimicrobial-resistant bacteria to three major classes of antimicrobials used in clinical medicine [29], [49], [61]. The spatial limitation of the present study hampers an immediate generalization of its conclusions to other aquacultural and control sites in Chilean coastal waters. Additional studies are thus necessary to confirm these results and to identify the dynamics of these processes more carefully. The presence of resistance genes to these antimicrobials in marine bacteria and residual antimicrobials in the sediment suggests that the increase in antimicrobial resistance results from selection of bacteria in this environment. The cautionary principle also suggests that use of antimicrobials in salmon aquaculture in Chile needs to be controlled and reduced because this increase has the potential to generate other problems of food safety and industrial health [20], [22], [23], [71], [72]. We expect that the new sanitary scenario instituted in response to the epidemic of infectious salmon anemia will result in a significant reduction in antimicrobial use and particularly in avoidance of quinolone antimicrobials because of their relevance to human health [18], [23], [73].

## Materials and Methods

### Location of aquaculture and control sampling sites

The two sites studied were located in the Calbuco archipelago in southern Chile, Region X, near the town of Calbuco (41°48′S, 73°11′W) (Fig. 1). At least 11 salmon farming sites have been in operation in this area over the past 10 years with an estimated annual production of approximately 15,000 metric tons. One of these farms consists of two salmon culture units with 22 and 24 pens located approximately 300 m from the coastline at a water depth of 45 m (arrow, Fig. 1). It can produce approximately 1,200 metric tons of salmon annually. Because of a confidentiality agreement, neither the exact identity of the farm, the biomass of fish cultured, nor the identity and amounts of antimicrobials used before and during the period of study can be revealed. Areas directly under the salmon pens are protected by anti-predator nets and are inaccessible to divers. For this reason, sediment samples from this site (“aquaculture site”) were obtained from an area approximately 20 m from the outer southeastern corner of the pens next to a buoy (arrowhead, lower inset of Fig. 1). This distance (<50 m) and the amount of organic matter at this site (>3.5%, unpublished data), indicates that its sediments were influenced by aquacultural activity [74], [75]. The other site (“control site”) was located 250 m off the northern coast of Tabón Island at a water depth of 30 m (Fig. 1). Tabón Island is 6 km long and close to 200 m wide and is situated 8 km south of the aquaculture site. It is the only local island without aquacultural activities, has few human dwellings, no sources of water and lacks solid discharges into the sea. Water currents at both sites vary between 15 and 18 cm s^−1^ during tidal flood and ebb, respectively, suggesting a high dispersal of materials in the water column and from sediments at both sites. Water surface temperature and salinity in this area vary from 9°C and 28‰ (parts per thousand) in the winter to 18°C and 32‰ in the summer. Both sampling sites are located in open access areas for which entrance and sampling permits are not required by Chilean regulations.

Sediment samples were taken from a circumscribed area of superficial sediment at the aquaculture and control sites by scuba divers using 15 cm diameter PVC plastic core samplers. After the sediment had been obtained, the sampler was closed by the diver with a plastic cap to avoid contamination. No endangered or protected organisms were captured in course of obtaining these sediment samples. The dates for sampling were arbitrarily chosen to encompass a full year with an emphasis on sampling during the austral spring/summer when aquacultural activities are concentrated. Sampling was performed seven times over a 12 month period: September, November and December, 2008; and January, April, July and September, 2009. Three samples were taken in September, 2008; five samples were taken at each of the other times resulting in 33 samples from the aquaculture site and 33 samples from the control site for a total of 66 samples from both sites. In November 2008, five samples were also taken at sites 0.5 km and 1 km from the aquaculture site for a total of 10 samples from these additional sites (Fig. 1).

### Measurement of antimicrobials in sediment samples

The presence in sediment samples of oxytetracycline, oxolinic acid, flumequine, and florfenicol was determined at the Instituto de Farmacia, Universidad Austral, Valdivia, Chile, by HPLC using standard protocols at fixed UV/Vis wave lengths [60], [76], [77]. These assays were done on four sediment samples taken from the aquaculture site and four sediment samples taken from the control site on each of four dates: December, 2008, January, 2009, April, 2009, and July, 2009. Two sediment samples each taken 0.5 and 1 km from the aquaculture site in November, 2008, were also tested for these antimicrobials. A total of 36 samples were tested for antimicrobials.

### Bacterial cultures

Culturable antimicrobial-susceptible and -resistant bacteria were determined by suspending 0.1 g of the top 2-cm of each sediment sample in 0.9 ml phosphate buffered saline, pH 7.4, within 2–3 hours after collection; this suspension was then sonicated (Elma Transsonic 310, Singen, Germany) for 5 minutes to ensure detachment of bacteria from sediment particles. A 10-fold dilution series (undiluted to 10^−5^) of this suspension was plated on Marine agar plates (Difco) containing no antimicrobials or oxytetracycline, 150 µg ml^−1^ (AppliChem GmbH, Darmstadt, Germany), or oxolinic acid, 10 µg ml^−1^ (Sigma-Aldrich GmbH, Steinheim, Germany), or florfenicol, 30 µg ml^−1^ (Sigma-Aldrich GmbH). Stock solutions of antimicrobials were kept frozen at −20°C and thawed immediately before use. Plates were incubated for 7 days at 20°C and the number of visible colonies were counted. These data were used to calculate cfu g^−1^ sediment = (total plate counts×dilution factor), and ARF (in percent) for each antimicrobial = (total plate counts with antimicrobial×dilution factor/total plate counts without antimicrobial×dilution factor)×100 [38]. Isolated colonies of bacteria growing on Marine agar plates with and without antimicrobials were selected and stored frozen at −80°C in 96-well microtiter plates in 36% glycerol for later study of antimicrobial resistance genes.

### Detection of plasmid-mediated antimicrobial resistance genes

Cultures of marine sediment samples obtained from December, 2008, and January, 2009, were transported in Marine soft agar in 1.5 ml Eppendorf microtubes and restreaked on Marine agar (9.0 cm diameter Petri dishes) containing antimicrobials to ascertain clonality. One isolated colony of each was grown for further studies and stored in 30% glycerol at −80°C. Bacterial cultures were kept at 4°C on marine agar plates with antimicrobials for daily manipulations while experiments were in progress. The following antimicrobial resistance genes were studied by PCR using primers shown in Table 3. Quinolone resistance genes: topoisomerase protection *qnrA*, *qnrB*, *qnrC*, *qnrD* and *qnrS* genes [40], [78]–[80]; the putative enzymatic inactivation gene *aac(6′)-Ib-cr* [40], [63]; and efflux pump genes *qepA* and *oqxA* [81], [82]. Tetracycline resistance genes: efflux pump genes *tetA*, *tetB* and *tetK* [64], [65], [83]; and the ribosome protection gene, *tetM* [64], [65]. Florfenicol resistance gene, *floR*, encoding an efflux pump [66], [84]. The *int1* gene encoding integrase 1 [41], [45] was also examined. Table 3 also contains information regarding strains used as positive and negative controls in these PCR reactions. Single colonies of oxytetracycline, oxolinic acid, and florfenicol resistant strains were picked from plates kept at 4°C and inoculated into 10 ml Marine Broth (Difco, BD, Franklin Lakes, NJ, USA) with ciprofloxacin HCl (ICN, Aurora, Ohio, USA), 0.05 µg ml^−1^, oxytetracycline hydrochloride (Sigma-Aldrich, St. Louis, MO, USA), 150 µg ml^−1^, or florfenicol (Sigma-Aldrich, St. Louis, MO, USA), 30 µg ml^−1^, and cultured at 20°C until late log phase. Cultures were centrifuged at 200× g for 15 min to remove suspended matter. The supernatant was centrifuged at 7,000× g for 10 min to pellet the bacteria, pellets were washed once with phosphate buffered saline, pH 7.4, and resuspended in 400 µl 10 mM TrisCl-10 mM EDTA for storage at −80°C until DNA was extracted [85]. Total DNA was extracted from 200 µl of bacteria by adding 10% SDS in sodium phosphate buffer, pH 8.0, to 1.25% SDS (wt/vol), incubating at 70°C for 30 min, and then disrupting bacteria with three freeze-thaw cycles: two minutes liquid N_2_-10 min 70°C (once), two minutes liquid nitrogen-10 min 100°C (twice). After centrifugation of the viscous solutions at 4,000× g for 10 min, the supernatant was transferred to a clean tube used for PCR screening of antimicrobial resistance genes [86]. All PCR assays were done using MasterCycler Gradient, Eppendorf, Germany. Multiplex PCR for *qnrA/B/S* was conducted in 25 µl reaction volumes with GoTaq Flexi DNA polymerase, 0.8 units (Promega, Madison, WI, USA); 1× Green GoTaq Flexi Buffer; MgCl_2_, 2 mM; dNTP, 0.15 mM each; primers, 0.5 µM each. Initial denaturation at 95°C for 3 min was followed by 35 cycles of 95°C, 20 sec-54°C, 30 sec-72°C, 40 sec; final extension of 72°C-7 min. For detection of the other PMQR, *tet* and *floR* genes, reaction volumes were 12.5 µl, and PCR was performed with Choice TaqBlue DNA polymerase 0.5 units (Denville Scientific Inc., Metuchen, NJ); 1× reaction buffer with MgSO_4_, 15 mM; dNTP, 0.25 mM each; primers 0.5 µM each. Initial denaturation at 3 min at 95°C was followed by 35 cycles of denaturation at 95°C-20 sec; annealing for 30 sec at various temperatures for each group of primers; extension time at 72°C was dependent on fragment length, being 30 sec for a 500 bp fragment (see Table 3); final extension, 7 min-72°C. Amplicons were detected in 1% agarose gel with ethidium bromide, viewed and recorded in an Alpha Imager AIC, Alpha Innotech, Japan. Identity of amplicons was ascertained by comparison with positive controls and by DNA sequencing (GENEWIZ, Inc, South Plainfield, NJ, USA) of at least one amplicon of each gene (data not shown). DNA sequences were identified by BLAST analysis against the non-redundant nucleotide sequence database at GenBank.

**Table 3:** Primers used in this study.

Gene	Primer	Sequence (5′→3′)	Amplicon (bp)	Positive control	References
*qnrA*	qnrA1ROB	ATTTCTCACGCCAGGATTTG	516	pMG252	[79]
	qnrA2ROB	GATCGGCAAAGGTTAGGTCA			
*qnrB*	qnrB1ROB	GATCGTGAAAGCCAGAAAGG	469	pMG298	[79]
	qnrB2ROB	ACGATGCCTGGTAGTTGTCC			
*qnrC*	qnrC-F	GGGTTGTACATTTATTGA	447	pDNA qnrC	[80]
	qnrC-R	TCCACTTTACGAGGTTCT			
*qnrD*	qnrD fw	CGAGATCAATTTACGGGGAATA	592	pDNA qnrD	[78]
	qnrD rv	AACAAGCTGAAGCGCCTG			
*qnrS*	qnrS1ROB	ACGACATTCGTCAACTGCAA	417	pMG306	[79]
	qnrS2ROB	TAAATTGGCACCCTGTAGGC			
*qepA*	qepA-F	CGTGTTGCTGGAGTTCTTC	403	pAT851	[82]
	qepA-R	CTGCAGGTACTGCGTCATG			
*oqxA*	oqxAF	CTCGGCGCGATGATGCT	392	DNA	[81]
	oqxAR	CCACTCTTCACGGGAGACGA			
*aac(6′)-Ib-cr*	Aac(6′)-1bXbaI	CAGCTCTAGAATTTTTAAGCGTGCAT	620	pMG298	[63]
	Aac(6′)-1b2R	ATATGCGAATTCTTAGGCATCACTGC			
*tetA*	tetAf_A3	GCCTCCTGCGCGATCTGG	848	pEDtetA2	[65]
	tetAr_A2	CGAAGCAAGCAGGACCATG			
*tetB*	tetB_BF	CAGTGCTGTTGTTGTCATTAA	571	pEDtetB1	[65]
	tetB_BR	GCTTGGAATACTGAGTGTAA			
*tetK*	tetKf	TCGATAGGAACAGCAGTA	169	pT181	[83]
	tetKr	CAGCAGATCCTACTCCTT			
*tetM*	tetM_M6	GTTTATCACGGAAGYGC	687	pJFP76	[65]
	tetM_M4	GAAGCCCAGAAAGGATTYGGT			
*floR*	flo_f	AATCACGGGCCACGCTGTATC	215	pAB5S9	[66]
	flo_r	CGCCGTCATTCTTCACCTTC			
*int1*	intI1F	GTTCGGTCAAGGTTCTGG	890		[45]
	intI1R	CGTAGAGACGTCGGAATG			
16S rDNA	16sRNAf	AGAGTTTGATCCTGGCTCAG	variable		[88]
	16sRNAr1	ACGGCTACCTTGTTACGACTT			

### Species identification of marine bacteria containing antimicrobial resistance genes

Identification of marine bacteria containing antimicrobial resistance genes was done by PCR amplification of 16S ribosomal genes [87], [88] using primers 16S rRNAf and 16S rRNAr1 (Table 3). The amplicons obtained were approximately 1500 bp and spanned 99% of 16S rRNA genes. Amplicons were sequenced and were identified by BLAST analysis against the non-redundant nucleotide sequence database at GenBank.

### Statistical analysis

To insure data quality and to exclude erratic values in quantitation of colonies the following three criteria were used. 1. If colony counts were consistent with a dilution series (roughly monotonic and decreasing with increasing dilutions), cfu g^−1^ were calculated using the plate with the lowest dilution with <160 colonies. 2. If colony counts were consistent with a dilution series but no plate had <160 colonies, cfu g^−1^ were calculated using the plate with the lowest dilution with >160 colonies. 3. If colony counts were not consistent with a dilution series (suggesting incomplete dispersal in the undiluted material), cfu g^−1^ were calculated using the plate with the lowest dilution consistent with a dilution series with <160 colonies. Data not meeting these criteria (5 of 258 points) were excluded from analysis. For computational purposes, plates with no colonies were imputed a value of 1, a value at the non-detect level. Data were analyzed by two-way ANOVA using rank transformed data [89] and a Student-Newman-Keuls post-hoc test as appropriate. Values of P≤0.05 were considered significant. A sensitivity analysis was done.
